# Learning Epithelial Elasticity via Local Tension Remodeling

**DOI:** 10.64898/2025.12.17.694921

**Published:** 2025-12-19

**Authors:** Sadjad Arzash, Shiladitya Banerjee

**Affiliations:** 1 School of Physics, Georgia Institute of Technology, Atlanta, GA 30332

## Abstract

Biological materials, like epithelial tissues, exhibit remarkable adaptability to mechanical stresses, dynamically remodeling their structure in response to external and internal forces. A key challenge is understanding how these tissues store a memory of past mechanical stimuli. Here, we investigate this memory using an active Vertex Model of epithelial sheets incorporating a local, mechanosensitive tension-remodeling rule where junctional tension updates depend on strain, acting as a slow, history-dependent variable. We demonstrate three hallmark mechanical consequences of this memory mechanism. First, a localized, short contractile cue permanently reprograms the global shear modulus, with the direction of change (stiffening or softening) controlled by the tension remodeling rate. Second, the tissue stores a long-range mechanical memory: a prior stimulus at one site modulates the tissue’s response to a subsequent, distant stimulus, mediated by coupling across the entire junctional network. Finally, we show that simple cyclic bulk deformation acts as a training protocol that autonomously tunes the tissue’s constitutive properties, including programming the Poisson ratio to auxetic (negative) values. These findings position epithelial mechanics within the framework of unsupervised physical learning, identifying the mechanosensitive remodeling rates as powerful control parameters for designing programmable tissue-scale rheology.

## INTRODUCTION

Many disordered materials achieve functional properties by storing memories of past stimuli [1-8]. Under repeated driving, these systems undergo irreversible microstructural changes so that their subsequent response encodes information about the training history [9-11]. A particularly relevant example is the “directed aging” protocol, in which disordered elastic networks are slowly driven while they relax, allowing local bond stiffnesses or rest lengths to evolve [2]. This algorithm can steer a network toward target mechanical tasks, such as producing an auxetic response, where the Poisson ratio is negative and the material expands laterally when stretched [12].

These ideas have recently been unified under a broader framework of “physical learning” in driven soft materials [13-19]. In this view, a physical system is described by fast dynamical degrees of freedom, such as node positions in an elastic network, coupled to slow internal variables, such as bond stiffnesses or rest lengths, that serve as tunable/learning parameters. Learning consists of updating these internal variables according to local rules, with the goal to optimize a task-dependent cost function [20] Because each update uses only locally accessible information, the training is decentralized and does not require computing global gradients, in contrast to standard gradient-descent optimization in parameter space.

Epithelial tissues are a particularly important case study for this paradigm, representing biological materials that inherently possess adaptive, learning capabilities. As active, confluent sheets, their junctional tension, adhesion, and cortical forces are dynamically remodeled by both external and internal cues [21-29]. While passive mechanical models of epithelial tissues (which rely on fixed parameters) can reproduce baseline viscoelastic spectra and geometry-driven rigidity transitions [30-32], they are unable to capture history-dependent changes in stiffness, spatial patterning in force transmission, or mechanosensitive remodeling observed *in vivo* [33-35]. These biological processes demand material adaptation, making mechanosensitive remodeling rules essential for epithelial mechanics.

Adaptive models bridge this gap, explicitly placing epithelia within the physical learning framework. For instance, allowing cell shapes to tune as additional degrees of freedom shifts and controls rigidity transitions, while local, length- and orientation-dependent updates of edge tensions reproduce adaptive tissue flows during convergent extension[36, 37].

Critically, unlike supervised learning protocols, biological systems, such as epithelial tissues, do not employ an external cost function or protocol for adaptation. Their remodeling rules are driven intrinsically by local biophysical feedback mechanisms [34]. This places epithelial tissues in the category of unsupervised physical learning, where the system’s material properties are autonomously programmed by its inherent adaptive rule, rather than by a pre-designed task or global minimization scheme. We hypothesize that the mechanosensitive tension-remodeling rule, inherent to epithelial junctions, is sufficient for implementing unsupervised physical learning in tissue monolayers, enabling the encoding of long-range mechanical memory and the exhibition of autonomous programmability of global constitutive properties.

Building on our previously introduced framework of mechanical remodeling in epithelial junctions [28, 33, 34, 38, 39], we consider a model in which fast mechanical variables, such as cell vertex positions and cell pressures, relax quickly, while slower junctional tensions record memory through local, strain-dependent rules. Specifically, each junction’s tension is updated selectively in response to strain thresholds, with distinct rates for contraction ($k_{C}$) and extension ($k_{E}$). The ratio of these rates, $k_{C}∕k_{E}$, serves as the key control parameter for the tissue’s mechanical programming.

We find that a short, localized contractile cue can rewire the tissue’s macroscopic mechanical properties; the shear modulus increases or decreases depending on $k_{C}∕k_{E}$, highlighting this ratio as a sensitive control parameter for tissue-scale stiffening or softening. Furthermore, after an initial signal and full mechanical relaxation, subsequent stimuli at distant sites elicit responses distinct from naïve tissues, demonstrating a history-dependent, long-range memory stored in the junctional network. Finally, subjected to small-amplitude, cyclic bulk driving, the tissue’s mechanical response becomes trainable, with the Poisson’s ratio tunable to auxetic (negative) values when $k_{C}>k_{E}$. Conceptually, these findings position epithelial mechanics alongside other trainable materials: slow, local parameter adaptations, directed by rapid, globally driven fields, yield targeted changes without centralized optimization.

## METHODS

### Epithelial vertex model with mechanosensitive tension remodeling

We model epithelial tissue dynamics using a modified version of the standard two-dimensional vertex model [30, 40-42]. In this framework, each cell is represented as a polygon, and the degrees of freedom are the positions of the vertices shared between neighboring cells. The mechanical energy of the tissue is given by

(1) $$E=\sum_{\alpha =1}^{N}[K_{A}{\left(A_{\alpha }-A_{0}\right)}^{2}]+\sum_{\langle ij\rangle }\Lambda_{ij}\ell_{ij}+\Gamma_{a}\ell_{ij}^{2},$$

where the first summation runs over all cells, and the second over all edges between adjacent cells. Here, $A_{\alpha }$ and $A_{0}$ are the current area and the target area of cell α, and the coefficient $K_{A}$ control the stiffness associated with area deformations. The second term describes the energy due to interfacial tension: each cell-cell junction ij of length $\ell_{ij}$ is associated with an active tension $\Lambda_{ij}(t)$, which evolves dynamically. The last term describes the energy due to actomyosin contractility, where $\Gamma_{a}$ is the contractile force per unit length of the edge [33, 38]. The total tension on edge ij thus becomes $T_{ij}=\Lambda_{ij}+2\Gamma_{a}\ell_{ij}$. This equation implies that the tension along a junction, arising from actomyosin contractility, is proportional to its length. This models the postiive feedback effect that myosin recruitment increases as junction length increases [43]. Position $r_{i}$ of vertex i evolves in time as: $\mu {\dot{r}}_{i}=-\partial E∕\partial r_{i}$, where μ is the vertex friction coefficient.

**FIG. 1.**
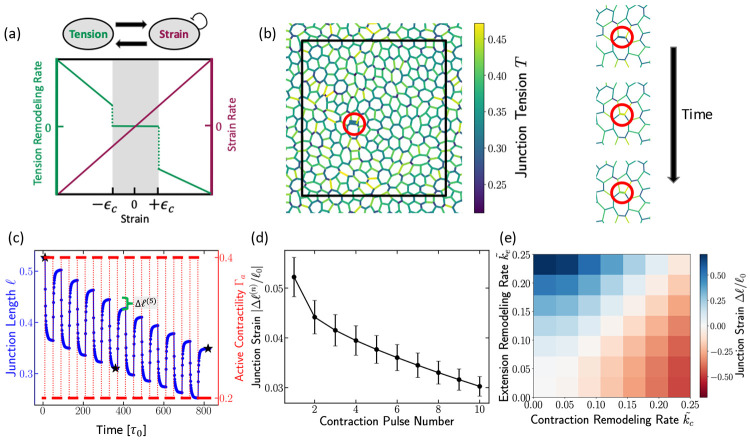
Adaptive dynamics of single junctions. (a) Schematic of the tension-remodeling model. In the contracted regime ($\varepsilon <-\varepsilon_{c}$), edge tension increases at a rate $k_{C}$ while in the extension regime ($\varepsilon >\varepsilon_{c}$), the tension decreases at a rate $k_{E}$. The edge strain relaxes continuously at a rate $k_{L}$. (b) A randomly selected edge in the tissue, with the colorbar indicating edge tensions. On the right: Three zoomed-in snapshots highlight the same edge at different times during application of a contractile active signal; the corresponding time points are marked with stars in panel (c). (c) Time series of the selected edge length (blue) under pulsatile contractile activations (red), illustrating how the edge progressively shortens with each activation. (d) Net reduction in edge length as a function of the number of activations. Although repeated activations further reduce the edge length, the incremental reduction diminishes with each sequential activation, akin to habituation. (e) Phase diagram of junctional strain as a function of ${\tilde{k}}_{C}$ and ${\tilde{k}}_{E}$ after the cessation of the contractile signal shown in red in panel (c).

Several recent studies have shown that intercellular tension in epithelial junctions is not fixed but continuously regulated by mechanochemical feedback [24, 28, 33-35]. To capture this adaptive behavior, we use the tension–remodeling model recently developed by one of us [33, 38], which quantitatively reproduces experimental observations of optogenetically driven junction contractions [33, 34]. In this framework, the tension $\Lambda_{ij}$ along each cell–cell junction evolves in time according to its strain,

(2) $$\frac{d\Lambda_{ij}}{dt}=-k(\varepsilon_{ij})(\ell_{ij}-\ell_{ij}^{0})-\frac{1}{\tau_{\Lambda }}(\Lambda_{ij}-\Lambda_{0}),$$

where $\ell_{ij}$ is the instantaneous junction length, $\ell_{ij}^{0}$ is its rest length, and the strain is $\varepsilon_{ij}=\left(\ell_{ij}-\ell_{ij}^{0}\right)∕\ell_{ij}^{0}$. The second term describes tension relaxation to its equilibrium value $\Lambda_{0}$ over a timescale $\tau_{\Lambda }$. The tension remodeling rate $k(\varepsilon_{ij})$ depends on the strain as (see Figure 1a)

(3) k(εij)={kC,εij<−εc,kE,εij>εc,0,otherwise,}

where $\varepsilon_{c}$ is a local threshold strain for junction remodeling. For positive $k_{C}$ and $k_{E}$, tension increases under contraction at rate $k_{C}$ and decreases under extension at rate $k_{E}$. The threshold $\varepsilon_{c}$ reflects the experimental observation that junctions remodel only above a critical strain amplitude, corresponding to sufficiently strong or sustained force [28, 33].

In addition to tension remodeling, cellular junctions continuously relax strain through rest-length remodeling. The rest length $\ell_{ij}^{0}$ of each junction evolves toward its current length $\ell_{ij}$ at a rate $k_{L}$ (see Figure 1a),

(4) $$\frac{d\ell_{ij}^{0}}{dt}=k_{L}(\ell_{ij}-\ell_{ij}^{0}),$$

representing the gradual turnover of strained actomyosin structures and their replacement by unstrained ones. This relaxation process naturally arises from cytoskeletal turnover and myosin exchange within junctional networks [33].

An important consequence of Eq. 4 is that the tissue gradually forgets prior deformations over the characteristic timescale $k_{L}^{-1}$. Sustained contractions therefore remodel junctions only up to a finite limit, as strain is progressively relaxed, whereas pulsatile contractions interspersed with rest periods enable cumulative, irreversible shortening through ratcheting [28, 33]. This interplay between tension remodeling and strain relaxation determines how long-term mechanical memory is written and erased in the tissue.

We perform our simulations using the open-source cellGPU package [44]. In the simulations, we nondimensionalize the equations of motion by setting $K_{A}=1$, $A_{0}=1$, and μ=0.1. Lengths are measured in units of $\sqrt{A_{0}}$, forces in units of $K_{A}A_{0}^{3∕2}$, and time in units of the characteristic timescale $\tau_{0}=\mu ∕(K_{A}A_{0})$ With our parameter choices, one simulation time unit corresponds to a physical timescale of order ~ 14s [38]. To initialize the tissue, we place N random seed points in a square periodic box of size $L_{x}=L_{y}=\sqrt{N}$ and construct the corresponding planar tissue using a Voronoi tessellation. To generate the initial tissue configuration, we first construct a confluent tiling governed by the energy $E=\sum_{\alpha =1}^{N}[K_{A}{\left(A_{\alpha }-A_{0}\right)}^{2}+K_{P}{\left(P_{\alpha }-P_{0}\right)}^{2}]$, where $A_{\alpha }$ and $P_{\alpha }$ are the area and perimeter of cell α, and $A_{0}$ and $P_{0}$ are their target values [30, 31]. We set $K_{A}=K_{P}=1$, $A_{0}=1$, and $P_{0}=3.7$, and obtain a mechanically equilibrated state by minimizing E with respect to vertex positions. The resulting force-balanced configuration serves as the reference tissue. We then initialize the edge tensions $\Lambda_{ij}$ in our adaptive vertex model to match the tensions computed from this equilibrium configuration. We then run the overdamped dynamics of (1) together with tension dynamics (2) and rest-length dynamics (4) until the system reaches a steady state with uniform active tensions, $\Lambda_{ij}=\Lambda_{0}$. This state serves as the initial tissue configuration for all subsequent simulations.

During all simulations, T1 transitions are triggered when an edge length falls below a prescribed cutoff value (see SI for details). In the results presented below, we work in the limit of slow tension relaxation compared to tension remodeling, $\tau_{\Lambda }^{-1}\ll k\sqrt{A_{0}}∕\Lambda_{0}$. The effect of faster relaxation timescales $\tau_{\Lambda }$ is explored in detail in the Supporting Information, and its implications for the robustness of mechanical memory are discussed in the main text.

## RESULTS

### Single junction mechanics and memory encoding

We first elucidate the mechanism of mechanical memory encoding at the scale of a single cell-cell junction, which is crucial for the tissue’s emergent collective behavior. To this end, we examine the mechanical response of a single junction subjected to a pulsatile $\Gamma_{a}(t)$ (Fig. 1 b,c), simulating pulsatile contractions that are widely observed during morphogenesis [45-49]. In the tension–remodeling model, these periodic stimuli produce a clear ratcheting effect: each pulse shortens the junction and the post-pulse length remains below its pre-pulse value, consistent with epithelial junction experiments [34]. Mechanistically, when the instantaneous strain crosses the negative threshold in (3), the update rule in (2) increases the junctional tension during the contracted phase. After the external contractility is removed, the junction relaxes toward a longer length, but the elevated tension (the stored memory) prevents full recovery, yielding a net shortening (Fig. 1c).

Successive pulses further increase tension and accumulate shortening, while the incremental shrinkage per pulse decreases over time (Fig. 1d), reflecting adaptation of the junctional state. This diminishing response under repetitive stimuli is a signature of *habituation*, a simple form of non-associative learning [50]. In this context, habituation demonstrates that the junction possesses memory: the stored tension from previous pulses modifies the system’s current mechanical response, leading to a permanent, adapted state. This process validates the local tension-remodeling rule as a mechanism for encoding and accumulating mechanical memory.

The direction and magnitude of the permanent length change ($\Delta \ell ∕\ell_{0}$) are controlled by the two dimensionless tension remodeling rates, ${\tilde{k}}_{C}=k_{E}∕k_{L}$ (contraction remodeling rate) and ${\tilde{k}}_{E}=k_{E}∕k_{L}$ (extension remodeling rate). This ratio acts as the fundamental control parameter for local mechanical adaptation (Fig. 1e). When the contraction remodeling rate dominates (${\tilde{k}}_{C}>{\tilde{k}}_{E}$), pulses drive a net shortening. Whereas, when the extension remodeling rate dominates (${\tilde{k}}_{E}>{\tilde{k}}_{C}$), the same contractile pulses can surprisingly produce a net lengthening of the junction. In the SI, we show how an edge successively lengthens under a pulsatile contractile signal in the regime $\tilde{k}>{\tilde{k}}_{C}$. This behavior is summarized in the phase diagram of the junctional strain $\Delta \ell ∕\ell_{0}$ (Fig. 1e), which delineates the shortening and lengthening regimes in the (${\tilde{k}}_{C},{\tilde{k}}_{E}$) plane. The ratio ${\tilde{k}}_{C}∕{\tilde{k}}_{E}$ determines the direction of the final structural change. Moreover, the net edge length change depends sensitively on the pulse period: for short periods the length change is negligible, whereas increasing the period produces larger changes that eventually saturate at a plateau value for this signal (see Fig. S2 in the SI). Based on experimental data [33, 51], we expect epithelial tissues typically operate in the regime where the contraction-induced remodeling rate is higher (${\tilde{k}}_{C}>{\tilde{k}}_{E}$), favoring net shortening and stiffening. The single-junction ratcheting and habituation establish the microscale learning rule that enables the emergent, collective mechanical reprogramming studied in the subsequent sections.

### Local cues reprogram global elastic property

**FIG. 2.**
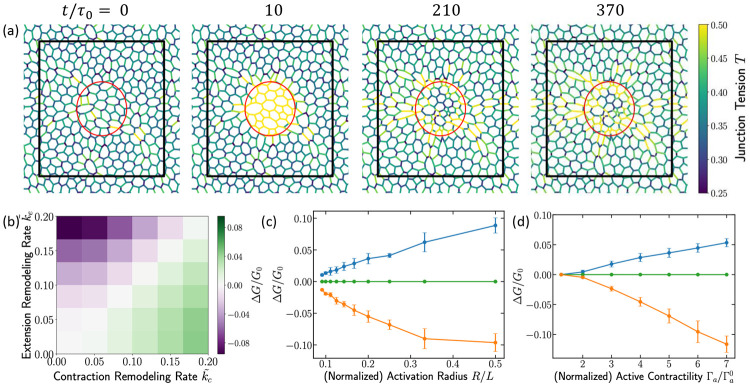
Local junction remodeling reprograms global mechanical properties. (a) Simulation snapshots of a circular activation region of radius R at the tissue center. The colorbar indicates edge tensions. The first snapshot shows the initial configuration with no contractile signal. The second snapshot shows the response during activation, where edges within the region develop high tension. The remaining snapshots illustrate the relaxation after the signal is removed, revealing that high-tension edges persist and spread into surrounding cells. These dynamics demonstrate that a locally applied contractile signal propagates through the tissue via feedback between tension and strain fields. (b) Phase diagram of the change in tissue shear modulus after five consecutive contractile activations applied to a central region of size R∕L=1∕6. The applied signal matches that of Fig. 1, with $\Gamma_{a}^{0}=0.2$ (low contractility), $\Gamma_{a}=1.2$ (high contractility), and pulse duration 40. (c) Change in shear modulus at $\Gamma_{a}∕\Gamma_{a}^{0}=4$ as a function of region size R∕L. Blue: ${\tilde{k}}_{C}=0.2$, ${\tilde{k}}_{E}=0$; green: ${\tilde{k}}_{C}={\tilde{k}}_{E}=0$; orange: ${\tilde{k}}_{C}=0$, ${\tilde{k}}_{E}=0.2$. (d) Change in shear modulus at R∕L=1∕6 as a function of the contractility ratio $\Gamma_{a}∕\Gamma_{a}^{0}$. Color scheme as in (c).

We next ask whether localized, pulsatile contractility can reprogram tissue-scale material properties, demonstrating the macro-scale consequence of the junctional mechanical memory established previously. We apply contractile pulses to a circular region of radius R (Fig. 2a) and track how local junctional remodeling alters the tissue’s global mechanical response (Movie S1).

Localized contraction pulses initiate a sequence of events that generates non-local changes in tissue properties via memory propagation. First, junctions within the activated region contract, and through the process of tension remodeling and strain relaxation, they increase their active tension and permanently reduce their rest length. These local changes encode a mechanical memory of the stimulus in the junctional network. This local contraction then induces strain in the neighboring cell junctions. Because tension remodeling is mechanosensitive, these strained neighboring junctions undergo tension remodeling, which, in turn, alters their tension and rest length, propagating a mechanical signal well beyond the initial activated region via tension and rest-length dynamics (Fig. 2a). The permanent, history-dependent changes accumulated across the entire network result in a stable, reprogrammed mechanical state, which we quantify by measuring the linear shear modulus (G) of the fully relaxed tissue. This is done by applying an infinitesimal simple shear and evaluating the second derivative of the energy at zero strain (see SI text for details).

We report the change in shear modulus, $\Delta G=G_{\text{final}}-G_{\text{initial}}$, after a fixed number of active contractile pulses. The direction of the global change (ΔG) is determined by the imbalance of the local remodeling rates, ${\tilde{k}}_{C}$ and ${\tilde{k}}_{E}$, as shown in the phase diagram in Fig. 2b. This ratio serves as the global control knob for programming the tissue’s stiffness. Specifically, for ${\tilde{k}}_{C}>{\tilde{k}}_{E}$, contraction-side remodeling dominates, and the tissue exhibits stiffening (ΔG>0). Conversely, for ${\tilde{k}}_{E}>{\tilde{k}}_{C}$, extension-side remodeling dominates, leading to tissue softening (ΔG<0). In the passive limit where remodeling is absent (${\tilde{k}}_{C}={\tilde{k}}_{E}=0$), ΔG=0, confirming that the observed changes arise solely from tension remodeling. Since real epithelial tissues are observed to be in the regime ${\tilde{k}}_{C}>{\tilde{k}}_{E}$ [33, 34], we predict a strain-stiffening behavior under localized active pulses. This offers a concrete, testable hypothesis using optogenetic tools combined with rheological experiments.

The magnitude of ΔG is highly tunable. Under a fixed number of pulses, ∣ΔG∣ increases approximately linearly with the size of the activated region for small R∕L (Fig. 2c). At fixed R, ∣ΔG∣ also grows with pulse amplitude $\Gamma_{a}$(Fig. 2d). Thus, localized contractions provide a control knob to program global mechanical properties, stiffening or softening, by tuning the balance of remodeling rates and the spatial extent and strength of the driven region.

### Long-range mechanical memory drives cooperative response

Having established that local tension remodeling enables mechanical memory encoding and can reprogram global stiffness, a key question is how this memory is communicated across the tissue, especially over distances exceeding cell size.

**FIG. 3.**
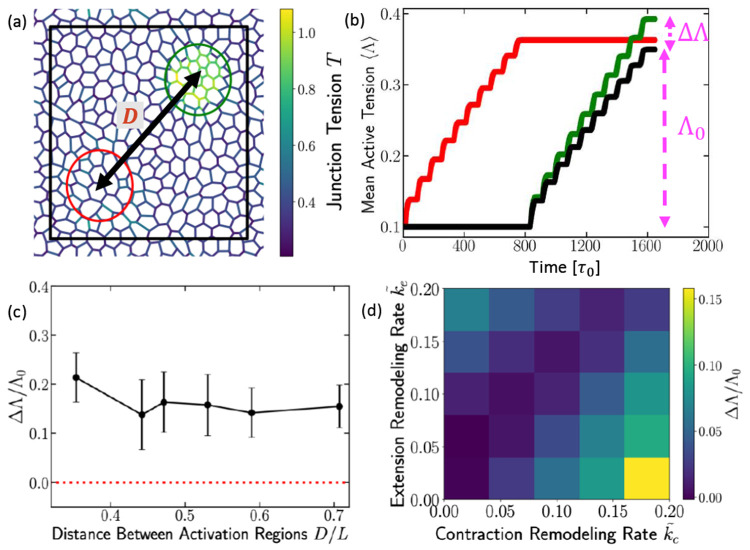
Long-range cooperative effects from local contractile signals. (a) Simulation snapshot showing contractile activations applied to two separate circular regions of the tissue: the first region (red) and the second region (green), each with radius R∕L=1∕6. The applied signal matches that of Fig. 1, with $\Gamma_{a}^{0}=0.5$, $\Gamma_{a}=2$, and pulse period 40. (b) Average active tension in the first region (red) and in the second region (green) during activation. The black curve shows the average active tension in the second region when the first region was not previously activated. (c) Change in the average active-tension in the second region, computed as the difference between the final values of tensions of the green and the black curves in (b). We refer to this as the *cooperative enhancement* which is plotted as a function of the center-to-center distance between these two active regions. (d) Cooperative enhancement phase diagram as a function of ${\tilde{k}}_{C}$ and ${\tilde{k}}_{E}$.

To address this, we designed a two-stage simulation. We first applied a localized contraction pulse to a ”trained” region (Region 1) of the tissue and allowed the system to mechanically relax. We then applied a second, identical pulse to a distant, ”test” region (Region 2), separated by distance D (Fig. 3a, Movie S2). We quantify the memory effect by comparing the average active tension generated in Region 2 under two conditions: (1) when Region 1 was previously activated (green curve, Fig. 3b) versus (2) when Region 1 was naive (black curve, Fig. 3b). The results show that the average tension in Region 2 rises more rapidly and reaches a higher final value when the tissue has previously experienced an active contraction elsewhere (Fig. 3b). We define the cooperative enhancement (ΔΛ) as the difference in the final average active tension (Λ) in Region 2 due to the prior activation of Region 1 (Fig. 3b). This non-local change indicates a long-range mechanical memory effect, not previously appreciated in eptihelial mechanics.

To determine the spatial extent of this memory, we systematically varied the center-to-center distance D between the two activated regions (Fig. 3a). As shown in Figure 3c, the memory effect (ΔΛ) decays slowly with increasing distance D but critically saturates to a finite, non-zero value. This suggests that the interaction is not purely local, such as through direct cell-cell contact, but is instead mediated through long-range mechanical coupling in the whole tissue. The permanent tension and rest-length changes stored locally in Region 1 globally bias the elastic response of the tissue, making the distant Region 2 more susceptible to remodeling upon activation.

The strength of this cooperative effect further depends on the rates of contraction (${\tilde{k}}_{C}$) and extension (${\tilde{k}}_{E}$) induced tension remodeling (Fig. 3d). This dependence shows that the ${\tilde{k}}_{C}∕{\tilde{k}}_{E}$ ratio not only dictates the direction of adaptation but also amplifies the magnitude of the long-range memory effect. Specifically, for the experimentally relevant regime where ${\tilde{k}}_{C}>{\tilde{k}}_{E}$, increasing ${\tilde{k}}_{C}$ at a fixed ${\tilde{k}}_{E}$ increases the cooperative enhancement (ΔΛ) (Fig. 3d). This amplification occurs because the initial stimulus (Region 1 activation) creates a large number of high-tension, memory-encoding junctions. A higher ${\tilde{k}}_{C}$ ensures that the local tension accumulated in these junctions is greater and more stable, resulting in a globally stiffer initial state for the tissue. When Region 2 is then activated, the enhanced stiffness transmits a stronger baseline stress through the network, pushing the local strain in Region 2 more quickly past the remodeling threshold and accelerating its own tension accumulation.

### Programming auxeticity via cyclic deformations

Having established that tissues can adapt under local contractile activity, we next ask whether such adaptive dynamics can be harnessed to design materials with targeted macroscopic properties. A particularly useful property to train is the Poisson’s ratio (ν), which quantifies the transverse response of a material under uniaxial strain. Materials with a negative Poisson’s ratio (auxetic materials) expand laterally when stretched and contract laterally when compressed. These desirable auxetic responses, which enhance shear resistance and energy absorption, are actively pursued in the design of mechanical metamaterials and architected solids [12, 52-54]. Recent studies have shown that periodic mechanical training, cyclically deforming disordered networks, can tune their internal structure to produce auxetic behavior through local remodeling [12]. Here we show that epithelial-like adaptive networks can achieve the same effect autonomously, through mechanosensitive tension remodeling at cell junctions.

**FIG. 4.**
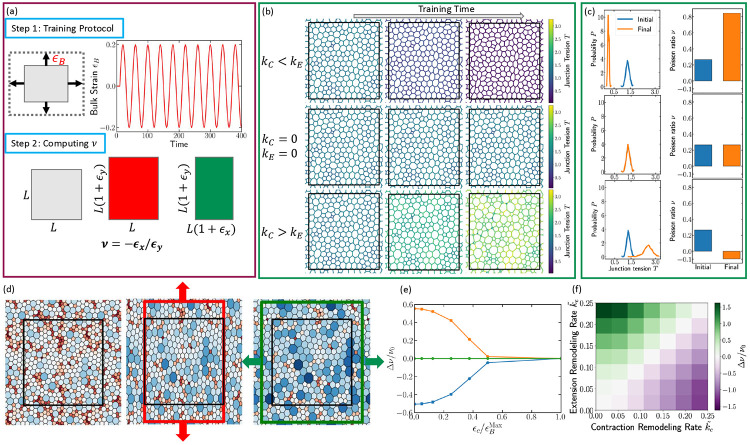
Training auxetic states under periodic bulk deformations. (a) Training tissue’s Poisson ratio under sinusoidal bulk deformations. Upper left panel: schematic of a bulk deformation with magnitude $\varepsilon_{B}$. Upper right: sinusoidal bulk-deformation signal applied over time. Lower panel: Schematic of the method used to compute the Poisson ratio. (b) Tissue snapshots under bulk deformation. Top row: evolution from the initial state to mid-training and the final configuration for ${\tilde{k}}_{C}<{\tilde{k}}_{E}$. Middle row: case with ${\tilde{k}}_{C}=\tilde{k}=0$. Bottom row: case with ${\tilde{k}}_{C}>{\tilde{k}}_{E}$. (c) Initial (blue) and final (red) edge-tension distributions for the three cases shown in panel (b). The right three plots show the corresponding initial (blue) and final (red) Poisson ratios for each scenario. (d) Auxetic response of a tissue under uniaxial stretch. Cells are colored by their area deviation, $\Delta A_{\alpha }=A_{\alpha }-A_{0}$, with blue indicating larger-than-rest areas and red indicating smaller-than-rest areas. Left: undeformed reference state (black rectangle). Middle: tissue under an imposed deformation along the *y*-direction (red arrows and red rectange). Right: relaxed configuration showing lateral expansion of the *x*-boundary (green arrows and green rectangle), consistent with a negative Poisson ratio. For visual clarity, this example uses a relatively large applied deformation; all reported values of the linear Poisson ratio ν are computed using a small strain of 1%. (e) Change in Poisson ratio as a function of the ratio of the critical edge-level strain to the maximum applied bulk strain, $\varepsilon_{c}∕\varepsilon_{B}^{\text{Max}}$. Orange: ${\tilde{k}}_{C}=0$, ${\tilde{k}}_{E}=0.2$; green: ${\tilde{k}}_{C}={\tilde{k}}_{E}=0$; blue: ${\tilde{k}}_{C}=0.2$, ${\tilde{k}}_{E}=0$. (f) Phase diagram for the case $\varepsilon_{c}=0.01$ and $\varepsilon_{B}^{\text{Max}}=0.2$.

To test this programmability, we subject the tissue to externally imposed oscillatory bulk deformations of amplitude $\varepsilon_{B}$ (Fig. 4a, Movie S3). During each cycle of isotropic compression and expansion, all cell edges experience local strain, while the adaptive variables, edge tensions $\Lambda_{ij}$ and rest lengths $L_{ij}$, evolve according to (2) and (4). These deformations act as a mechanical training protocol that gradually tunes the microscopic edge properties and, consequently, the emergent elastic response of the tissue. After applying a fixed number of bulk-training cycles, we relax the system to mechanical equilibrium and measure its effective Poisson’s ratio. To compute ν, we impose a small uniaxial strain $\varepsilon_{y}$ along the *y*-axis, allow the tissue to relax, and measure the induced transverse strain $\varepsilon_{x}$ along the *x*-axis. The Poisson’s ratio is then calculated as $\nu =-\varepsilon_{x}∕\varepsilon_{y}$ (Fig. 4a).

Figure 4b shows representative tissue configurations and tension fields for three regimes: (1) $k_{C}<k_{E}$, (2) $k_{C}=k_{E}=0$, and (3) $k_{C}>k_{E}$. When $k_{C}<k_{E}$, edge tensions decrease over repeated bulk cycles, leading to more uniform cell areas (see SI for area distributions) and an increase in ν. In the passive control model without remodeling ($k_{C}=k_{E}=0$), both tensions and ν remain unchanged. By contrast, when $k_{C}>k_{E}$, edge tensions grow monotonically during training, generating strong structural and tension heterogeneity with coexisting large and small cells (Fig. 4b,c). In the SI, Fig. S3 shows that the area distribution in this regime broadens substantially, with distinct populations of very small and very large cells. Fig. S4 of SI shows that cell pressure distributions narrow and shift to low pressures for $k_{C}<k_{E}$ but remain broad, with both negative and positive pressures, for $k_{C}>k_{E}$. This highly tensed, heterogeneous architecture drives ν downward, ultimately yielding negative values characteristic of an auxetic response (Fig. 4c).

The physical origin of this auxetic behavior is a rigidity switch controlled by the competition between junctional tension and cell area elasticity. In the extension-dominated regime (${\tilde{k}}_{E}>{\tilde{k}}_{C}$), junction tensions are low, and the stiff area elasticity term ($K_{A}{\left(A_{\alpha }-A_{0}\right)}^{2}$) dictates the mechanical response. To maintain cell area when stretched along the *y*-axis, the tissue contracts laterally, resulting in a positive Poisson ratio (ν>0). Conversely, in the regime ${\tilde{k}}_{C}>{\tilde{k}}_{E}$, edge tensions increase continuously, and the tension energy term becomes dominant. In this highly tensed state, the cell area deformations becomes effectively softer than the tension network. When subjected to uniaxial stretch ($\varepsilon_{y}>0$), the vertices surrounding the stretched cells move outward in the transverse direction, causing lateral expansion ($\varepsilon_{x}>0$) to relieve the high internal tension. This outward motion yields the required negative Poisson ratio (ν<0) (see Fig. 4d).

The ${\tilde{k}}_{C}∕{\tilde{k}}_{E}$ ratio acts as the primary learning bias, as shown in the phase diagram (Fig. 4f), which demonstrates that ν can be tuned upward or downward by varying this ratio. Additionally, the training is gated by the ratio of the applied strain to the local remodeling threshold ($\varepsilon_{B}∕\varepsilon_{c}$). When the applied deformation $\varepsilon_{B}$ is too small, the tissue is inert; as the ratio $\varepsilon_{B}∕\varepsilon_{c}$ increases, adaptive remodeling becomes stronger, and the magnitude of the programmed change in ν grows rapidly (Fig. 4e).

These results demonstrate that epithelia-like adaptive networks can be trained through repeated bulk deformations to acquire programmable elastic responses, including negative Poisson’s ratios, without any designed geometry or plastic flow, purely through dynamic mechanosensitive feedback. In the regime $k_{C}>k_{E}$, our theory suggests that under oscillatory external deformations, tissues would continuously stiffen and decrease their Poisson ratio. This presents an intriguing experimentally testable prediction for epithelial monolayers.

## DISCUSSION

A minimal, mechanosensitive tension–remodeling rule is sufficient to produce three hallmark features of trained materials in epithelial sheets: history-dependent adaptation of global moduli, long-range mechanical memory, and learned elastic responses under cyclic driving. The same local update rule that captures optogenetic single-junction behavior [28, 33] reprograms tissue-scale elasticity. In particular, repeated bulk deformations tune the Poisson ratio, including into the auxetic regime, without architected geometry or plastic hinges, demonstrating a functional parallel with the programmability and history dependence observed in directed-aging and periodic-training strategies for disordered solids [12, 55]. This places epithelial mechanics within a broader physical-learning framework [20] in which fast elastic fields drive slow, local parameter updates that accumulate history and alter subsequent response.

The model identifies two primary control levers for programming the material. First, the imbalance between contraction- and extension-side remodeling sets the learning bias and the direction of training. When contraction-driven remodeling rate is higher ($k_{C}>k_{E}$), localized pulses stiffen the material and cyclic bulk driving lowers ν, switching the material to auxetic behavior. When extension-induced remodeling rate is higher ($k_{E}>k_{C}$), the opposite trends occur. Second, the amplitude of the applied strain relative to a local strain threshold for junctions ($\varepsilon_{B}∕\varepsilon_{c}$) gates learnability: for $\varepsilon_{B}<\varepsilon_{c}$, the epithelial sheet is inert; above threshold, per-cycle updates add coherently and the trained response grows.

Our main results are based on the limit of negligible tension relaxation ($\tau_{\Lambda }^{-1}\to 0$), assuming stable, persistent memory storage. To confirm that the observed phenomena are indeed contingent upon this memory, we analyzed the effect of finite tension relaxation timescale on the tissue’s adaptive response (SI Fig. S6). We find that memory-based effects, including the change in shear modulus (ΔG), the cooperative long-range response (ΔΛ), and the change in Poisson ratio (Δν), are all diminished when tension relaxation is fast compared to junction remodeling rate ($\tau_{\Lambda }^{-1}\gg \left(k\sqrt{A_{0}}\right)∕\Lambda_{0}$), where k denotes the strain-dependent remodeling rate ($k=k_{C}$ for contractile strains and $k=k_{E}$ for extensional strains). Only when tension relaxation is slow does the tissue retain a significant, persistent memory and exhibit programmed mechanical changes. This suggests that the remodeling rule constitutes a functional storage mechanism, as the rapid erasure of $\Lambda_{ij}$ eliminates all history-dependent mechanical responses.

Our work proposes new possible experimental tests on epithelial tissues. The tissue global rheology should be programmable by varying the radius and strength of the activated region: the change in shear modulus is predicted to scale approximately linearly with the size of the activation zone for small R∕L and to grow with pulse amplitude, with the sign determined by $k_{C}∕k_{E}$. The observed long-range mechanical memory also suggests a concrete experimental test: a local input pulse should bias the response to a later, distant pulse after full relaxation, revealing history-dependent coupling stored in cell junctional states. The magnitude of this bias should decay with separation before saturating, reflecting elastic coupling across the sheet. Moreover, under small-amplitude isotropic stretch–compression cycles, the Poisson ratio should change monotonically over training time and reach negative values when $k_{C}>k_{E}$.

These behaviors can be probed using optogenetic myosin activation tests and Poisson’s ratio measurements under cyclic training [24, 28, 56, 57]. The predicted tissue stiffening and shift toward auxetic behavior under cycling aligns with concurrent experimental work showing that cyclic loading increases epithelial tissue lifetime and tolerance to large deformations [57]. Measuring the change in Poisson ratio before and after such cyclic loading in epithelial sheets would provide a direct, comprehensive experimental test of our adaptive remodeling mechanism.

## Supplementary Material

1
