# Supplementary material for: Learning Epithelial Elasticity via Local Tension Remodeling
Source: bioRxiv. 2025 Dec 19:2025.12.17.694921. Preprint. [Version 1] doi: 10.64898/2025.12.17.694921 (PMC12724603; doi:10.64898/2025.12.17.694921)
Supplement: 1 [file NIHPP2025.12.17.694921v1-supplement-1.pdf]

bioRxiv preprint doi: https://doi.org/10.64898/2025.12.17.694921; this version posted December 19, 2025. The copyright holder for this preprint (which was not certified by peer review) is the author/funder, who has granted bioRxiv a license to display the preprint in perpetuity. It is made available under aCC-BY 4.0 International license.

SUPPLEMENTARY INFORMATION

Initial tissue configuration

To generate the initial tissue geometry, we use the open-source `CellGPU` package [44]. We place N random points as cell centers in a square simulation box of lateral size $L = \sqrt{N}$ with periodic boundaries, and construct the corresponding cellular network using a Voronoi tessellation. This yields a disordered confluent tissue composed of polygons with various numbers of neighbors.

To obtain a mechanically relaxed reference state, we minimize the classical vertex-model energy

$$E = \sum_{\alpha} K_A (A_\alpha - A_0)^2 + K_P (P_\alpha - P_0)^2, \tag{S1}$$

with $K_A = K_P = 1$, $A_0 = 1$, and $P_0 = 3.7$, placing the system in the solid-like regime. Here A_α and P_α denote the instantaneous area and perimeter of cell α.

Each cell α is represented as an n_α-sided polygon with ordered vertices $\{\mathbf{r}_m\}_{m=1}^{n_\alpha}$ (with $\mathbf{r}_{n_\alpha+1} \equiv \mathbf{r}_1$). Its geometric measures are computed as

$$A_\alpha = \frac{1}{2} \sum_{m=1}^{n_\alpha} (r_{m+1,x} + r_{m,x})(r_{m+1,y} - r_{m,y}), \tag{S2}$$

$$P_\alpha = \sum_{m=1}^{n_\alpha} \ell_{m,m+1}, \qquad \ell_{m,m+1} = \|\mathbf{r}_{m+1} - \mathbf{r}_m\|. \tag{S3}$$

We then relax the tissue using the FIRE algorithm [58] to obtain the minimum-energy configuration. T1 transitions are allowed during this process and are triggered whenever an edge contracts below a threshold length $\ell_{T_1} = 0.05$.

Shear modulus calculation

To compute the shear modulus G of the dynamic tension–remodeling model, we first arrest all dynamical updates of the edge tensions Λ_{ij}. For a given tissue configuration, the system is then relaxed to a mechanically stable state by minimizing the tension–remodeling vertex-model energy

$$E = \sum_{\alpha=1}^{N} K_A (A_\alpha - A_0)^2 + \sum_{\langle ij \rangle} \Lambda_{ij} \ell_{ij} + \Gamma_a \ell_{ij}^2, \tag{S4}$$

where A_α is the area of cell α, ℓ_{ij} is the length of edge ij, and Λ_{ij} are the fixed (frozen) edge tensions at the moment dynamics are halted. The minimization is performed using the FIRE algorithm, following the same above procedure used for preparing relaxed initial states.

Once the mechanically stable configuration is obtained, we compute the shear modulus by evaluating the second total derivative of the energy with respect to an infinitesimal simple shear strain γ. For each configuration, we apply an affine simple shear of the simulation box with amplitudes $\gamma = -\Delta\gamma$, 0, $+\Delta\gamma$, updating all vertex positions accordingly. After each shear step, the vertex positions are relaxed using the FIRE algorithm while keeping Λ_{ij} fixed, and we record the corresponding energies $E(-\Delta\gamma)$, $E(0)$, and $E(+\Delta\gamma)$. The shear modulus is then obtained from a centered finite-difference approximation to the second derivative,

$$G = \frac{1}{L^2} \frac{E(+\Delta\gamma) - 2E(0) + E(-\Delta\gamma)}{\Delta\gamma^2}. \tag{S5}$$

We have verified that the resulting G is insensitive to the choice of a small $\Delta\gamma$, confirming that the calculation is performed in the linear elastic regime. We note that this numerical procedure is consistent with the standard definition of the linear shear modulus obtained from the Hessian of the system [59, 60]. Because the tension degrees of freedom are held fixed during these minimizations, this procedure isolates the instantaneous elastic response of a given trained state of the tissue.

Extension of an edge under contractile signals

In the main text, we showed that when $\tilde{k}_E > \tilde{k}_C$, an edge subjected to pulsatile contractile signals can surprisingly undergo a net extension over time. Figure S1 illustrates this behavior. During each contractile pulse, the edge initially shortens, as expected.

bioRxiv preprint doi: https://doi.org/10.64898/2025.12.17.694921; this version posted December 19, 2025. The copyright holder for this preprint (which was not certified by peer review) is the author/funder, who has granted bioRxiv a license to display the preprint in perpetuity. It is made available under aCC-BY 4.0 International license.

However, in the relaxation phase that follows, the influence of neighboring edges places the junction in a strain regime where $\varepsilon > \varepsilon_C$. In this regime, the tension–remodeling rule reduces the edge tension. Repeated cycles of contraction and relaxation therefore drive a progressive decrease in tension, leading to a net extension of the edge despite the applied contractile signals.

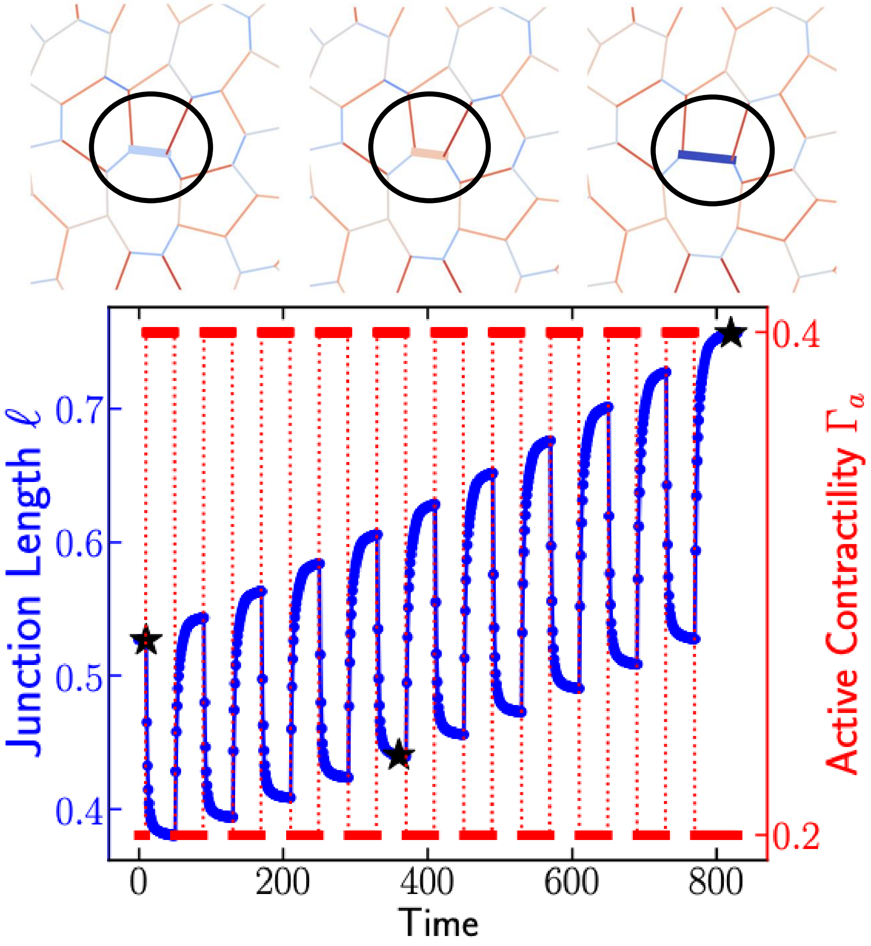

FIG. S1. Edge length (left, blue axis) and applied contractile signal (right, red axis) as functions of time in the regime $\tilde{k}_E > \tilde{k}_C$ (here $\tilde{k}_E = 0.2$ and $\tilde{k}_C = 0$). The top row shows consecutive snapshots of the edge corresponding to the time points marked with black stars in the main plot. Here, the junction-level critical strain is $\varepsilon_C = 0.1$.

Effect of active contractility period

The duration of the active contractile pulse strongly influences the edge response. Figure S2b shows the net change in edge length as a function of the contractility period $\mathscr{T}$. For very short periods, the edge has insufficient time to remodel, resulting in negligible length change. As $\mathscr{T}$ increases, the accumulated remodeling leads to a monotonic increase in length change, which eventually saturates to a plateau. This has been observed in experiments of epithelial junctions cite.

Structural features

Periodic bulk oscillations modify tissue structure through the adaptive coupling between junctional strain and tension. We quantify these structural changes by tracking the evolution of cell-scale geometric statistics after training. As shown in Fig. S3a, when $\tilde{k}_C > \tilde{k}_E$ cell areas develops a pronounced high-variance distribution, characterized by the simultaneous presence of cells with enlarged and shrunk areas. This increase in area heterogeneity results in the reduction in Poisson ratio reported in the main text. In contrast, when $\tilde{k}_C < \tilde{k}_E$, the area distribution narrows and concentrates around the mean area of 1, consistent with a more uniform confluent monolayer.

bioRxiv preprint doi: https://doi.org/10.64898/2025.12.17.694921; this version posted December 19, 2025. The copyright holder for this preprint (which was not certified by peer review) is the author/funder, who has granted bioRxiv a license to display the preprint in perpetuity. It is made available under aCC-BY 4.0 International license.

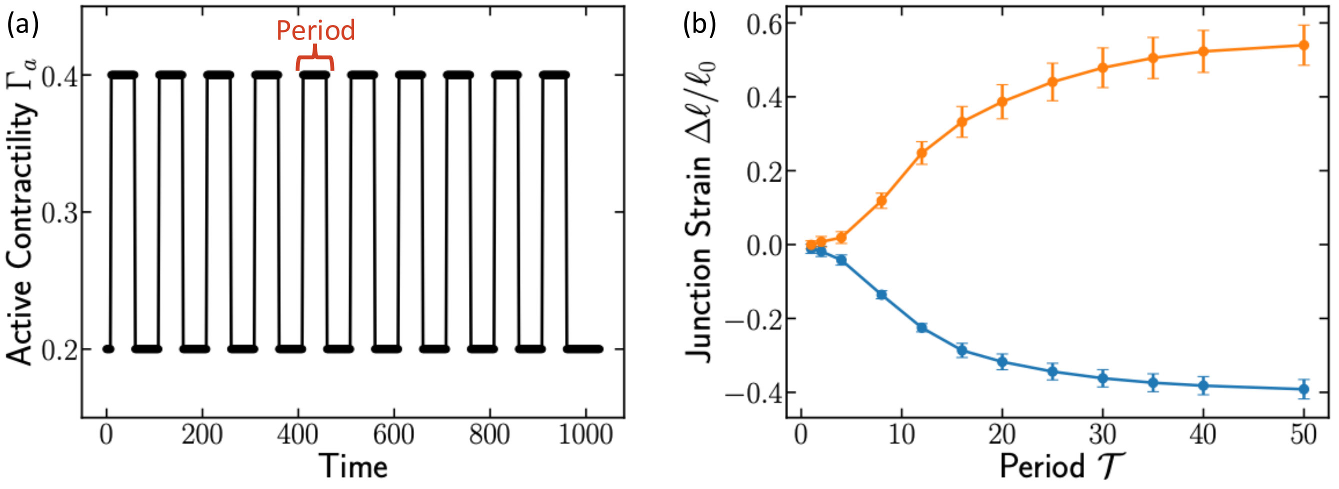

FIG. S2. (a) Pulsatile contractility signal alternating between a low value $\Gamma_a = 0.2$ and a high value $\Gamma_a = 0.4$. The period of this signal controls the extent of junctional remodeling. (b) Final change in junction length after applying the signal in (a) for different periods $\mathcal{T}$. The orange curve corresponds to the regime $\tilde{k}_E > \tilde{k}_C$ (here $\tilde{k}_E = 0.2$, $\tilde{k}_C = 0$), where edges exhibit net extension. The blue curve shows the regime $\tilde{k}_C > \tilde{k}_E$ (here $\tilde{k}_C = 0.2$, $\tilde{k}_E = 0$), where edges contract under repeated pulses.

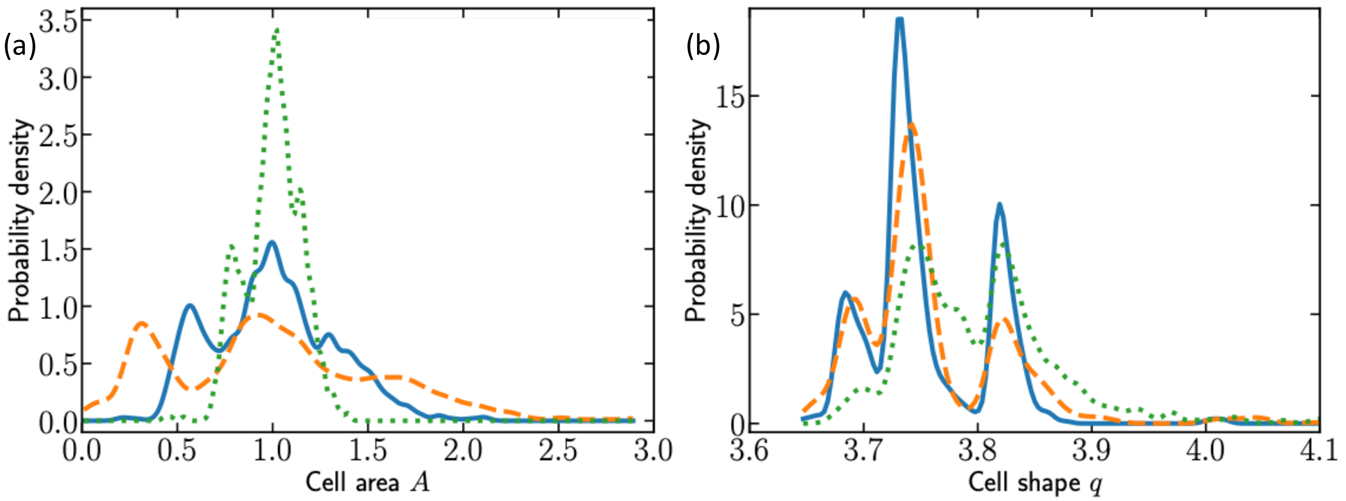

FIG. S3. (a) Probability density of cell areas. Shown are the initial distribution (solid blue), the post-training distribution for bulk–strain training with $\tilde{k}_C = 0.25$, $\tilde{k}_E = 0$ (dashed red), and the post-training distribution for $\tilde{k}_C = 0$, $\tilde{k}_E = 0.25$ (dotted green). (b) Corresponding distributions of the cell shape index $q = P/\sqrt{A}$ computed from the same configurations as in panel (a). The data is averaged over 5 different random samples.

Figure S4 shows the evolution of cell-level pressure distributions under bulk–strain training. For $\tilde{k}_C > \tilde{k}_E$, the distribution develops a pronounced both positive high-pressure and negative high-pressure tails, indicating the emergence of tiny and big cell populations as training progresses. In contrast, when $\tilde{k}_C < \tilde{k}_E$, cell pressures shift toward low but positive values, reflecting a more homogeneous cell area distributions. The resulting state is closer to a fluid-like, weakly compressed configuration, consistent with the increase in Poisson ratio reported in the main text.

Figure S5 shows the distributions of edge strain after bulk–strain training. The initial configuration has zero strain by construction. When $\tilde{k}_C > \tilde{k}_E$, the post-training distribution develops a pronounced heavy tail, indicating the presence of edges that undergo large relative elongation. In contrast, for $\tilde{k}_C < \tilde{k}_E$, the strain distribution remains sharply peaked, reflecting a population of edges that experience only small deviations from their reference lengths.

bioRxiv preprint doi: https://doi.org/10.64898/2025.12.17.694921; this version posted December 19, 2025. The copyright holder for this preprint (which was not certified by peer review) is the author/funder, who has granted bioRxiv a license to display the preprint in perpetuity. It is made available under aCC-BY 4.0 International license.

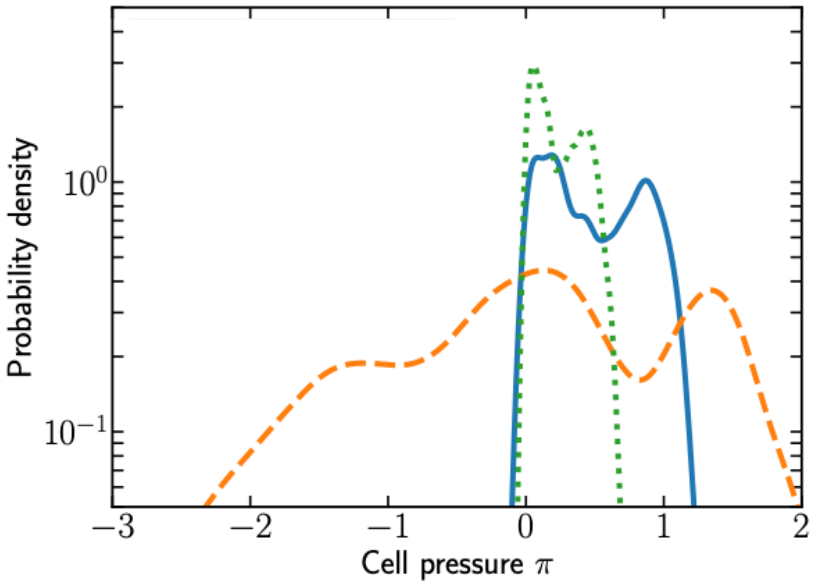

FIG. S4. Probability density of cell pressures $\pi_i = -2K_A(A_i - A_0)$ for the same configurations shown in Fig. S3. Displayed are the initial pressure distribution (solid blue), the post-training distribution for bulk–strain training with $\tilde{k}_C = 0.25$, $\tilde{k}_E = 0$ (dashed red), and the post-training distribution for $\tilde{k}_C = 0$, $\tilde{k}_E = 0.25$ (dotted green). Data are averaged over 5 independent random samples.

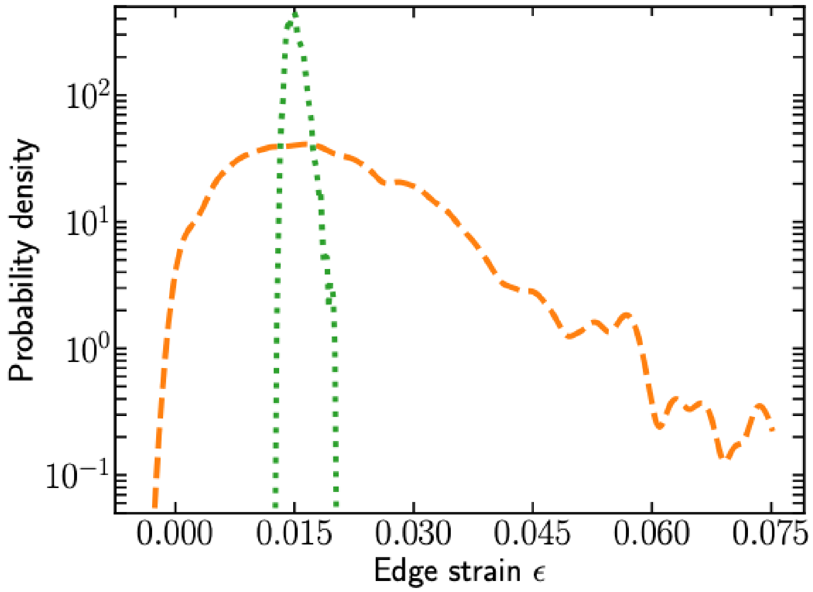

FIG. S5. Probability density of edge strains $\varepsilon_{ij} = (\ell_{ij} - \ell^0_{ij})/\ell^0_{ij}$ for the trained tissues shown in Fig. S3. Displayed are the post-training strain distributions for bulk–strain training with $\tilde{k}_C = 0.25$, $\tilde{k}_E = 0$ (dashed red) and for $\tilde{k}_C = 0$, $\tilde{k}_E = 0.25$ (dotted green). The initial configuration is not shown because all edges begin at zero strain. Data are averaged over 5 independent random samples.

Effect of finite tension relaxation

The tension–remodeling framework described in the main text generates clear signatures of mechanical memory and adaptive response. In biological tissues, however, junctional tension relaxes over a finite timescale due to actomyosin turnover and adhesion dynamics. Rapid relaxation should diminish the influence of prior loading, whereas slow relaxation preserves it. All results presented so far correspond to the limit of no relaxation, $\tau_\Lambda = \infty$.

bioRxiv preprint doi: https://doi.org/10.64898/2025.12.17.694921; this version posted December 19, 2025. The copyright holder for this preprint (which was not certified by peer review) is the author/funder, who has granted bioRxiv a license to display the preprint in perpetuity. It is made available under aCC-BY 4.0 International license.

To examine how finite relaxation alters the dynamics, we augment the tension update rule by adding a relaxation term:

$$\frac{d\Lambda_{ij}}{dt} = -k(\varepsilon_{ij})\left(\ell_{ij} - \ell_{ij}^0\right) - \frac{1}{\tau_\Lambda}\left(\Lambda_{ij} - \Lambda_0\right), \tag{S6}$$

where the first term is the strain-dependent remodeling introduced in the main text, and the second term drives Λ_{ij} toward a baseline value Λ_0 over a timescale τ_Λ.

To examine how tension relaxation shapes mechanical memory, we first measured how a localized contractile pulse alters the shear modulus. Figure S6a shows the resulting change in shear modulus as a function of the inverse relaxation timescale τ_Λ^{-1}. When relaxation is fast, the imposed tension rapidly returns to its baseline value and the tissue retains no memory of the pulse, leading to essentially no change in G. As relaxation slows (smaller τ_Λ^{-1}), the effects of the contractile event persist, resulting in a finite increase in the shear modulus. In the limit of very slow relaxation, $\tau_\Lambda^{-1} \to 0$, ΔG approaches the no-relaxation behavior described in the main text.

We next quantified how finite tension relaxation modifies the cooperative, long-range memory effect described in the main text. In the absence of relaxation, tissues that previously experienced a contractile pulse in one region respond more strongly to a subsequent pulse applied in a distant region, reflecting persistent changes in the underlying tension field. To measure this cooperative effect, we computed the difference between the final average tension in the second activated region when a prior contractile event occurred elsewhere and the corresponding tension when no prior activation was applied. Figure S6b shows the normalized response $\Delta\Lambda/\Lambda_0$ in the second region as a function of the inverse relaxation timescale τ_Λ^{-1}. When relaxation is fast (large τ_Λ^{-1}), the cooperative effect vanishes: tension rapidly returns to its baseline value, eliminating any imprint of earlier contractile signals. As relaxation slows (smaller τ_Λ^{-1}), the cooperative response increases, indicating the accumulation of a finite mechanical memory. In the limit of very slow relaxation, the effect saturates to a plateau corresponding to the no-relaxation regime.

Figure S6c shows how the Poisson's ratio changes in tissues trained under the bulk oscillation protocol described in the main text, plotted as a function of the inverse tension–relaxation timescale τ_Λ^{-1}. When tension relaxes rapidly larger (τ_Λ^{-1}), the system effectively erases the history of loading, and the final mechanical state remains unchanged. In contrast, slow relaxation allows tension updates to accumulate, producing a finite shift in tissue properties and a persistent trained state. In biological epithelia, tension relaxation is governed by actin turnover and myosin motor dynamics, which operate on finite timescales; as a result, tissues can maintain mechanical memory over repeated deformation cycles.

SUPPLEMENTARY MOVIES

Movie S1. Global effects of local contractions. Time-lapse of a tissue subjected to pulsatile active contractility ($\Gamma_a(t)$) in a circular region of radius R at the tissue center, in the regime $k_C > k_E$. Cell–cell junctions are colored by their total tension T_{ij}. The shear modulus G, computed under this activation, increases over time, illustrating the stiffening behavior described in the main text.

Movie S2. Cooperative effects of local contractions. Time-lapse of a tissue subjected to pulsatile active contractility ($\Gamma_a(t)$) in two circular regions of radius R separated by a distance D, in the regime $k_C > k_E$. Cell–cell junctions are colored by their active tensions Λ_{ij}. The movie also shows the time evolution of the average active tension in each region, highlighting the enhanced response in the second region due to prior activation of the first, as quantified in the cooperative enhancement analysis in the main text.

Movie S3. Training tissues under oscillatory bulk deformation. Time-lapse of a tissue subjected to a sinusoidal bulk deformation signal $\varepsilon_B(t)$ in the regime $k_C > k_E$. Cell–cell junctions are colored by their tension T_{ij}. During training, junctional tensions grow and the tissue develops a heterogeneous structure with coexisting very large and very small cells. The Poisson ratio ν decreases over successive cycles and becomes negative, demonstrating the emergence of auxetic behavior discussed in the main text.

bioRxiv preprint doi: https://doi.org/10.64898/2025.12.17.694921; this version posted December 19, 2025. The copyright holder for this preprint (which was not certified by peer review) is the author/funder, who has granted bioRxiv a license to display the preprint in perpetuity. It is made available under aCC-BY 4.0 International license.

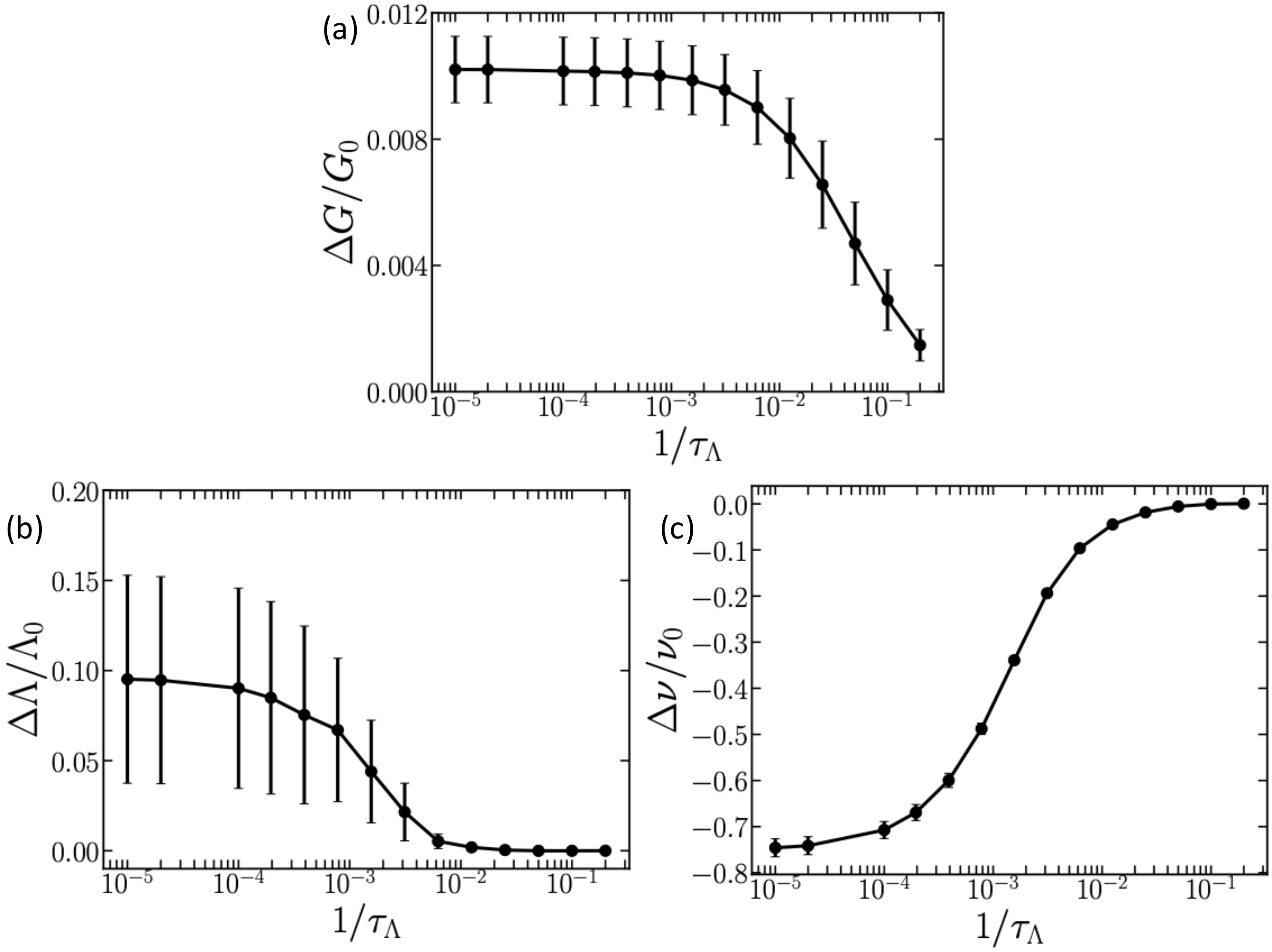

FIG. S6. (a) Normalized change in shear modulus $\Delta G/G_0$ as a function of the inverse tension–relaxation timescale τ_Λ^{-1}. The tissue is trained by applying a single contractile pulse in a central circular region of radius $R = L/6$ (with $L = \sqrt{N}$ and $N = 200$ cells). The active signal is increased from $\Gamma_a = 0.5$ to $\Gamma_a = 2.0$ for a duration $\mathscr{T} = 40$. Results are shown for contraction and extension remodeling rates $k_C = 0.2$ and $k_E = 0.05$. (b) Cooperative tension–memory response $\Delta\Lambda/\Lambda_0$ in a distant region as a function of τ_Λ^{-1}. The contractile signal is again pulsed between $\Gamma_a = 0.5$ and $\Gamma_a = 2.0$ for a duration $\mathscr{T} = 40$, but here applied repeatedly for 10 on–off cycles. The center–to–center separation between the previously activated region and the probe region is $D/L = 0.53$. The same remodeling parameters $k_C = 0.2$ and $k_E = 0.05$ are used. (c) Normalized change in Poisson's ratio as a function of the inverse tension–relaxation timescale τ_Λ^{-1}, for tissues trained under the bulk oscillatory protocol described in the main text. Results are shown for contraction and extension remodeling rates $\tilde{k}_C = 0.2$ and $\tilde{k}_E = 0.05$. The bulk strain amplitude for this training is $\varepsilon_B = 0.2$ and the edge-level critical strain is $\varepsilon_C = 0.01$.
